# Editorial: Primary Immunodeficiencies Worldwide

**DOI:** 10.3389/fimmu.2019.03148

**Published:** 2020-01-22

**Authors:** Menno C. van Zelm, Antonio Condino-Neto, Mohamed-Ridha Barbouche

**Affiliations:** ^1^Department of Immunology and Pathology, Central Clinical School, Monash University, Melbourne, VIC, Australia; ^2^Department of Respiratory, Allergy and Clinical Immunology (Research), Alfred Health, Melbourne, VIC, Australia; ^3^The Jeffrey Modell Diagnostic and Research Centre for Primary Immunodeficiencies, Melbourne, VIC, Australia; ^4^Department of Immunology, Institute of Biomedical Sciences, University of São Paulo, São Paulo, Brazil; ^5^The Jeffrey Modell Diagnostic and Research Center for Primary Immunodeficiencies, São Paulo, Brazil; ^6^Department of Immunology, Institut Pasteur de Tunis, University Tunis El-Manar, Tunis, Tunisia

**Keywords:** primary immunodeficencies (PID), intravenous immunoglobulin (IVIg), infectious disease, newborn screen (NBS), poliovirus (PV), Bacille Calmette-Guérin vaccine (BCG), developing and transition countries, consanguinity

The field of Primary Immunodeficiencies (PID) is growing fast. Over 400 diseases are defined and characterized by recurrent or chronic infections, autoimmunity, allergy, inflammation, or cancer as a consequence of genetic alterations affecting the immune system (1). The overall incidence of PID is around 1:10,000 and the majority presents early in childhood. Over the past decades, there has been an enormous increase in understanding of disease pathology, as well as clinical expertise and patient awareness worldwide, as a result of educational initiatives and scientific meetings by medical societies, supporting agencies, and patient associations.

Humans are genetically heterogeneous, and the environmental characteristics and social habits differ dramatically in the several geographic regions of the world. Consequently, the prevalence and distribution of the nine groups of primary immunodeficiencies vary worldwide, and the clinical profile of a particular PID will also vary.

Most of the literature and discoveries about Primary Immunodeficiencies come from North America and Europe and refer to these geographical areas. This Frontiers Research Topic was developed to bring together original clinical and basic research from authors representing various geographical areas to highlight challenges and developments in epidemiology, diagnosis, and treatment of Primary Immunodeficiencies worldwide.

Twenty-two articles are included in this Research Topic, and are categorized into the following types: 9 Original Research (Aghamohammadi et al.; de Albuquerque et al.; Goel et al.; Macklin et al.; Pietrucha et al.; Rechavi et al.; Schlechter et al.; Segundo et al.; Slade et al.), 2 Case Reports (Bradshaw et al.; Sogkas et al.), 2 Mini-reviews (Al-Mousa and Al-Saud; Barbouche et al.), 6 Reviews (de Jesus Nunes-Santos and Rosenzweig; Lee and Lau; Marciano and Holland; Ruffner et al.; Seleman et al.; Thakar et al.), 2 Perspectives (Jindal et al.; Quinti and Mitrevski), and 1 Opinion (Sorensen).

Within the context of epidemiology, several papers present and discuss infectious complications in international cohorts. It is discussed how cellular and molecular defects of the immune system predispose to invasive endemic fungal infections worldwide (Lee and Lau). Furthermore, it is shown that PID patients excrete poliovirus for extended periods (Macklin et al.), and that these may form a reservoir that forms a risk to poliovirus eradication strategies (Aghamohammadi et al.). Ruffner et al. provide a comprehensive overview of viral complications of PIDs. On top of infections, vaccination with live strains such as BCG are a major risk for complications in those countries where it is provided, including in India (Jindal et al.), and these complications are also associated with newly identified genetic causes of PID (de Jesus Nunes-Santos and Rosenzweig).

In addition to infectious complications, non-infectious complications are more frequently documented worldwide as exemplified in papers presenting the PID field in India (Jindal et al.), and an antibody-deficient cohort in Australia (Slade et al.). These complications most frequently involve autoimmunity, gastrointestinal disease, granulomatous inflammation, and malignancy. Disease complications vary even between individuals with the same genetic defect as illustrated by two siblings with RNF168 deficiency, highlighting roles for additional genetic or epidemiological factors (Pietrucha et al.). Countries in the Middle East and North Africa (MENA) with high rates of consanguinity face additional challenges (Al-Mousa and Al-Saud; Barbouche et al.), as some autosomal recessive disorders can be unexpectedly frequent and disease presentation can be complicated by other genetic changes.

Diagnostic challenges vary worldwide. Access to genetic genetics in developing countries is often limited, and shipment of patient material can be costly. The use of dried bloodspots for sequence analysis of genomic DNA will be a means to overcome the cost limitation (Segundo et al.). In developed countries, the implementation of next-generation sequencing technologies has taken flight (Seleman et al.), and facilitates quick diagnosis such as in the case of X-Linked Moesin-Associated Immunodeficiency (Bradshaw et al.) and MAP3K14 deficiency (Schlechter et al.).

The most optimal timing of PID diagnosis is before disease presentation and that is aimed at in newborn screening programs. A lot of experience has been obtained with the T cell receptor excision circle (TREC) assay in the USA (Thakar et al.), and many countries worldwide, including Israel, have now adopted it (Rechavi et al.).

Still, more insights into PID pathogenesis and genetics are needed. Diagnosis of antibody deficiency remains challenging, especially in the context of normal total IgG serum levels (Sorensen), and this can result in substantial diagnostic delays (Slade et al.). Functional assays for diagnostics require more insights into disease pathogenesis, and this lies at the heart of basic research into PID. Examples included in this Research Topic are examination of STAT phosphorylation upon cytokine receptor engagement (Goel et al.; Sogkas et al.), and *in vitro* models of innate immune responses to Candida (de Albuquerque et al.).

The ultimate focus of the Research Topic concerns treatment of PID. Immunoglobulin replacement therapy is already provided for over 60 years to supplement IgG, but has additional immunomodulatory roles that are still not completely understood (Quinti and Mitrevski), and might warrant the use even beyond antibody deficiency. More recently developed biologicals will hold even more promise over the coming years (Quinti and Mitrevski). Corrective therapy would be ultimate form of treatment, and hematopoietic stem cell transplantation is currently more widely applied in combined immunodeficiencies and in immune dysregulation disorders. Although the rarity of specific PIDs could hamper clinical trials, the genetic definition of disease should provide the best rationale for compassionate access to enable precision medicine.

## Author Contributions

MZ, AC-N and M-RB wrote the manuscript and approved of the final version.

### Conflict of Interest

The authors declare that the research was conducted in the absence of any commercial or financial relationships that could be construed as a potential conflict of interest. The handling editor declared a past co-authorship with one of the authors (AC-N).
